# Supplementary material for: Non-canonical functions of UHRF1 maintain DNA methylation homeostasis in cancer cells
Source: Nat Commun. 2024 Apr 5;15:2960. doi: 10.1038/s41467-024-47314-4 (PMC10997609; doi:10.1038/s41467-024-47314-4)
Supplement: Supplementary file 1 — Supplementary Information [file 41467_2024_47314_MOESM1_ESM.pdf]

Non-canonical functions of UHRF1 maintain DNA methylation homeostasis in cancer cells

Kosuke Yamaguchi*, Xiaoying Chen, Brianna Rodgers, Fumihito Miura, Pavel Bashtrykov, Frédéric Bonhomme, Catalina Salinas-Luypaert, Deis Haxholli, Nicole Gutekunst, Bihter Özdemir Aygenli, Laure Ferry, Olivier Kirsh, Marthe Laisné, Andrea Scelfo, Enes Ugur, Paola B. Arimondo, Heinrich Leonhardt, Masato T. Kanemaki, Till Bartke, Daniele Fachinetti, Albert Jeltsch, Takashi Ito, Pierre-Antoine Defossez*

* Authors for correspondence: yamako0801@icloud.com, pierre-antoine.defossez@cnrs.fr

List of Supplementary Information

Supplementary Figures

Supplementary Figure 1. Establishment and validation of endogenous AID-tagged UHRF1 and DNMT1 DLD1 cells and further validations and controls.

Supplementary Figure 2. Growth of DLD1 derivatives after UHRF1 or DNMT1 degradation; RNA-seq after auxin treatment, additional controls on the HCT116 derivatives and the rescue experiments.

Supplementary Figure 3. Validation of the effects of UHRF1 and DNMT1 degradation on DNA methylation in DLD1 cells; identification of the domains essential for DNA methylation.

Supplementary Figure 4. Greater loss of DNA methylation upon UHRF1 depletion than upon DNMT1 depletion; additional data.

Supplementary Figure 5. Additional controls for the UHRF1/DNMT3A/DNMT3B interaction; chromatome experiments reveal the effect of UHRF1 depletion on DNMT3B; validation of the DNMT3A/DNMT3B KOs.

Supplementary Figure 6. UHRF1 protects against active demethylation by TET2: additional data and controls.

Supplementary Tables

Supplementary Table 1. Summary of the primers and oligonucleotide sequences in this study.

Supplementary Table 2. Summary of WGBS basic metrics.

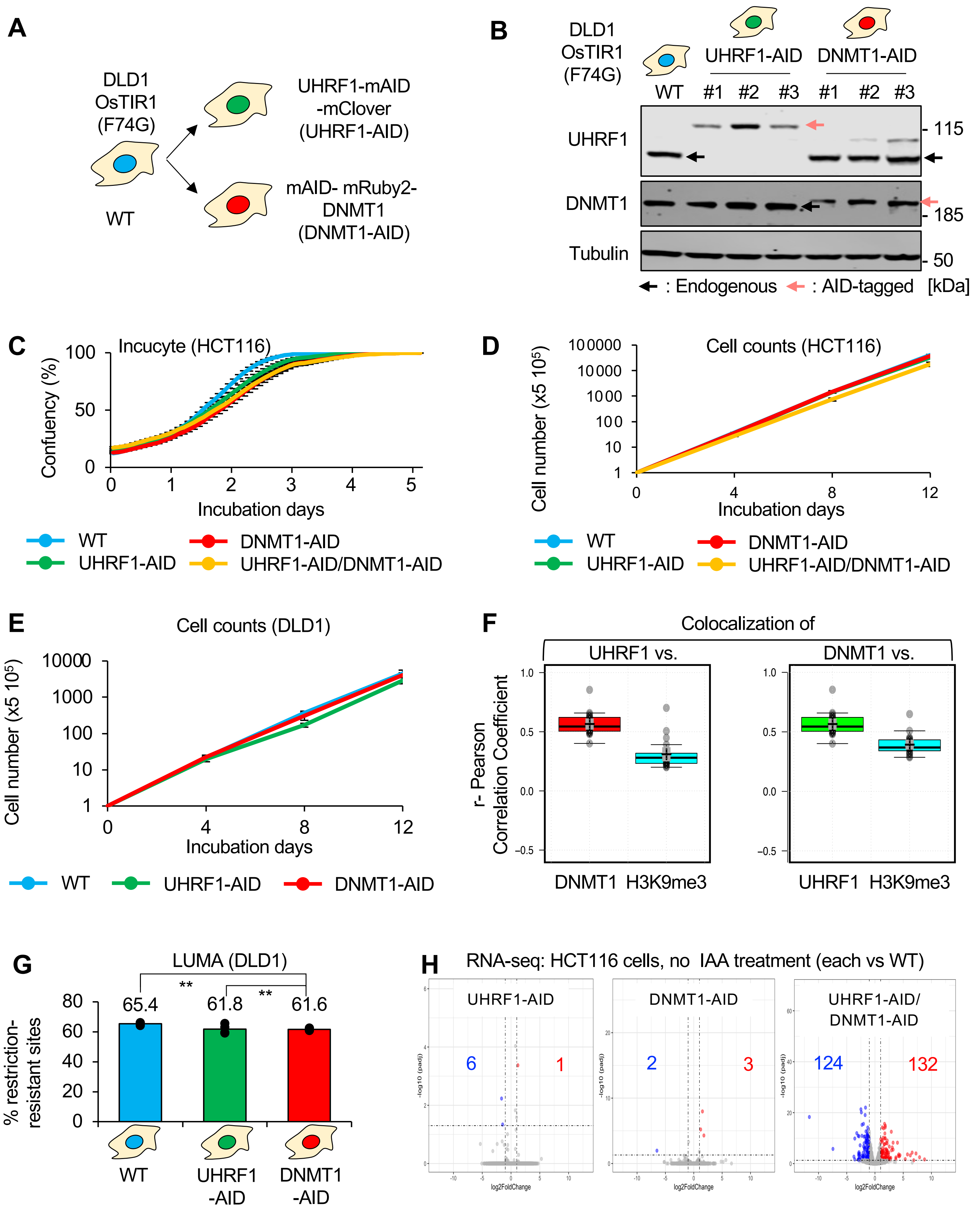

A
DLD1
OsTIR1
(F74G)
WT
UHRF1-mAID
-mClover
(UHRF1-AID)
mAID- mRuby2-
DNMT1
(DNMT1-AID)
B
DLD1
OsTIR1
(F74G)
UHRF1-AID
DNMT1-AID
WT
#1
#2
#3
#1
#2
#3
UHRF1
DNMT1
Tubulin
- 115
- 185
- 50
: Endogenous
: AID-tagged
[kDa]
C
Incucyte (HCT116)
Confluency (%)
Incubation days
WT
UHRF1-AID
DNMT1-AID
UHRF1-AID/DNMT1-AID
D
Cell counts (HCT116)
Cell number (x5 10^5)
Incubation days
WT
UHRF1-AID
DNMT1-AID
UHRF1-AID/DNMT1-AID
E
Cell counts (DLD1)
Cell number (x5 10^5)
Incubation days
WT
UHRF1-AID
DNMT1-AID
F
Colocalization of
UHRF1 vs.
DNMT1 vs.
r- Pearson
Correlation Coefficient
DNMT1
H3K9me3
UHRF1
H3K9me3
G
LUMA (DLD1)
% restriction-
resistant sites
65.4
61.8
61.6
**
**
WT
UHRF1
-AID
DNMT1
-AID
H
RNA-seq: HCT116 cells, no IAA treatment (each vs WT)
UHRF1-AID
6
1
DNMT1-AID
2
3
UHRF1-AID/
DNMT1-AID
124
132
log2FoldChange
-log10 (padj)

Supplementary Figure 1. Establishment and validation of endogenous AID-tagged UHRF1 and DNMT1 DLD1 cells and further validations and controls.

(A) Procedure followed to establish the UHRF1-AID and DNMT1-AID cells in the DLD1 background. (B) Immunoblot for validation of endogenous AID-tagged UHRF1 or DNMT1 DLD1 cells. Experiments in each panel were performed at least three times, and the representative results are shown. Experiments in each panel were performed at least three times, and the representative results are shown. (C) Cell proliferation data on HCT116 derivatives without auxin (Incucyte videomicroscopy). (D) Cell proliferation data on HCT116 derivatives without auxin (Cell counting). (E) Cell proliferation data on DLD1 derivatives without auxin (Cell counting). (F) The boxplots show the r- Pearson Correlation Coefficient of colocalization. Centre lines: medians; box limits: 25th and 75th percentiles; whiskers: 1.5 x interquartile range, bars indicate 95% confidence intervals of the means; 400 cells examined for each sample, all images corrected for aberrant chromatic shift. (G) Quantification of the DNA methylation level in DLD1 derivatives, without auxin (LUMA). The p-value is calculated with one-way ANOVA and Tukey's HSD test (**$p < 0.01$). Data are presented as mean values +/- SEM from three (n=3) biologically independent cells. (H) Differentially Expressed Genes (DEGs) between each cell line and WT, in the absence of auxin (IAA). Red dots: upregulated genes (Log2 Fold Change > 1, q-value < 0.01), blue dots: downregulated genes (Log2 Fold Change < -1, q-value < 0.01), gray dots: no significant change. The p-value is corrected to q-value using the Benjamini and Hochberg method.

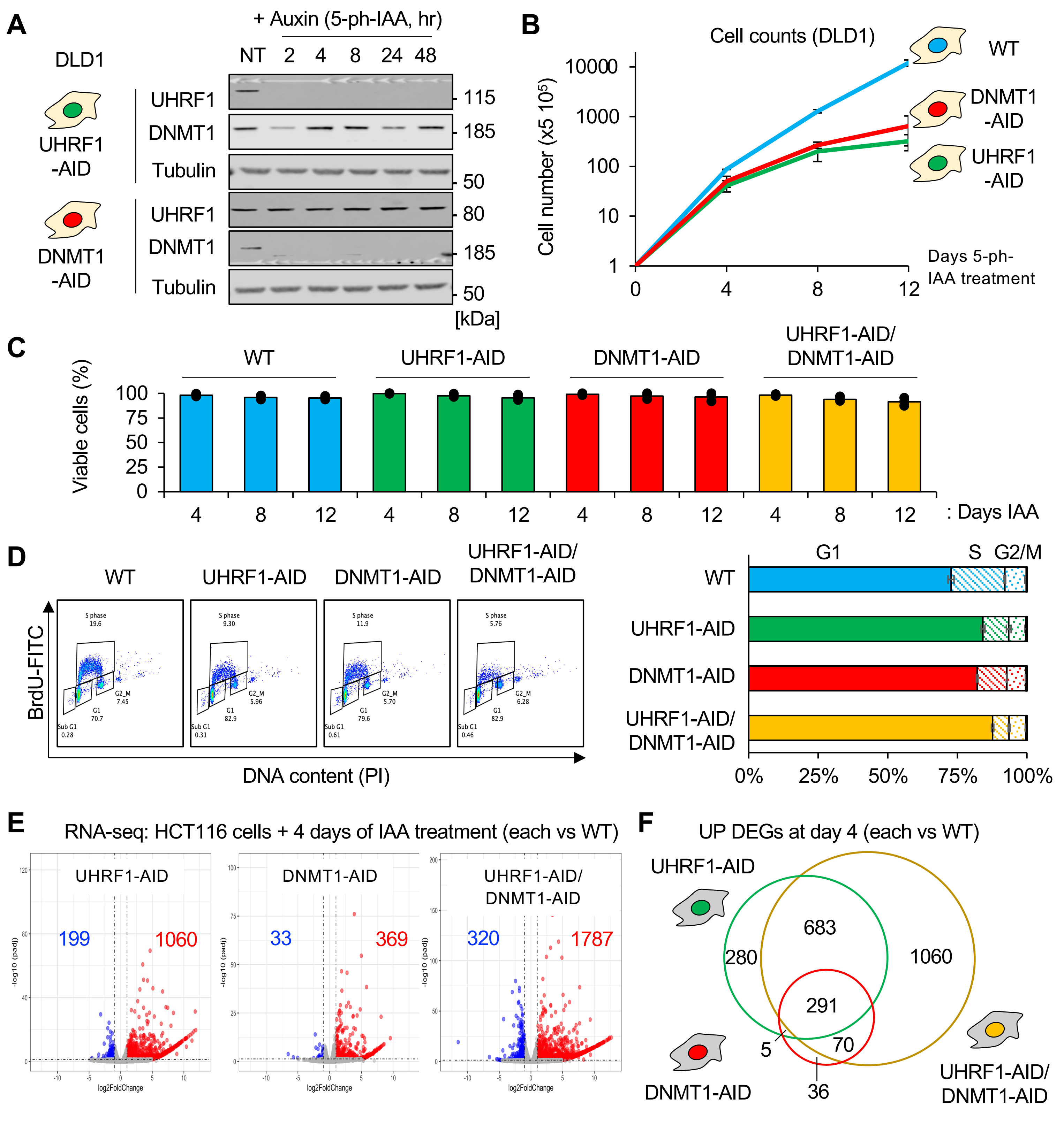

G

GSEA analysis	UHRF1-AID	DNMT1-AID	UHRF1-AID/ DNMT1-AID
Cancer/Testis gene set	NES=1.90, q=0.06	NES=1.69, q=0.11	NES=1.59, q=0.09
Hallmark E2F targets	NES=-2.78, q<0.001	NES=-2.35, q<0.001	NES=-2.68, q<0.001

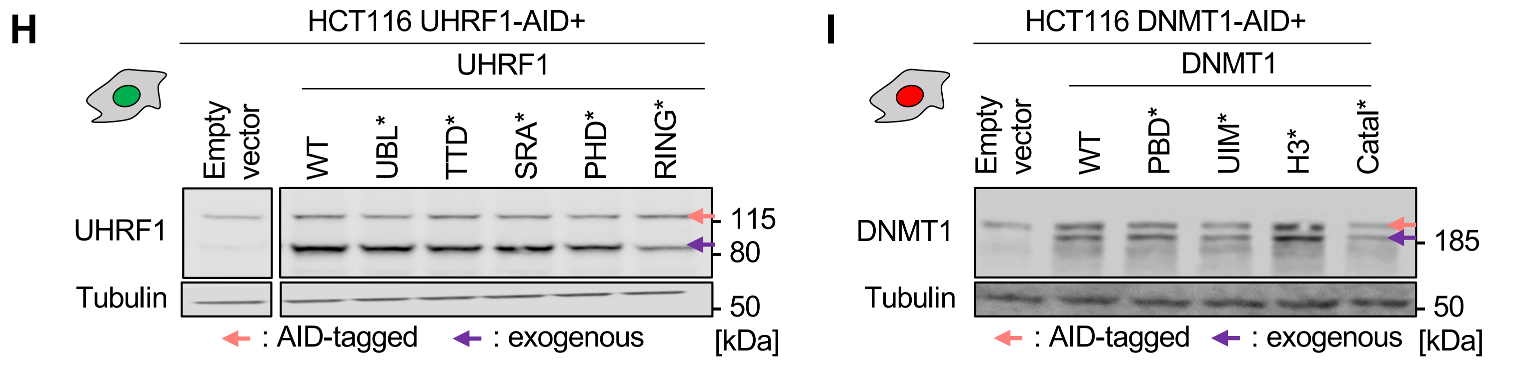

Supplementary Figure 2. Growth of DLD1 derivatives after UHRF1 or DNMT1 degradation; RNA-seq after auxin treatment, additional controls on the HCT116 derivatives and the rescue experiments.

(A) Immunoblot of DLD1 cells following treatment with Auxin at the indicated time points and before treatment (NT). (B) Cell proliferation of DLD1 derivatives after auxin addition. Error bars: SEM of biological triplicates. (C) Cell viability of HCT116 derivatives following UHRF1 or DNMT1 degradation (trypan blue staining). Error bars: SEM of biological triplicates. (D) Cell cycle analysis at day 4 of treatment with FACS. Left panel: Representative plot images for BrdU labeling FACS plot. The populations are assigned to the phases of the cell cycle as indicated. Right panel: Summary of the cell cycle population in each cell lines. Data are presented as mean values +/- SEM from biological triplicates. (E) Differentially Expressed Genes (DEGs) at day 4 of treatment. Red dots: upregulated genes (Log2 Fold Change > 1, q-value < 0.01), blue dots: downregulated genes (Log2 Fold Change < -1, q-value < 0.01), gray dots: no significant change. The p-value is corrected to q-value using the Benjamini and Hochberg method. (F) Venn Diagram for the UP DEGs. (G) GSEA analysis of the Differentially Expressed Genes. NES= Normalized Enrichment Score, q= FDR q-value. (H) Immunoblot of UHRF1 rescue constructs. The pink arrow indicates endogenous UHRF1 tagged with AID and mClover. The purple arrow indicates exogenous UHRF1 tagged with V5. Experiments in each panel were performed at least two times, and the representative results are shown. (I) Immunoblot images for validation of exogenous DNMT1 rescue constructs. Legend as in panel H.

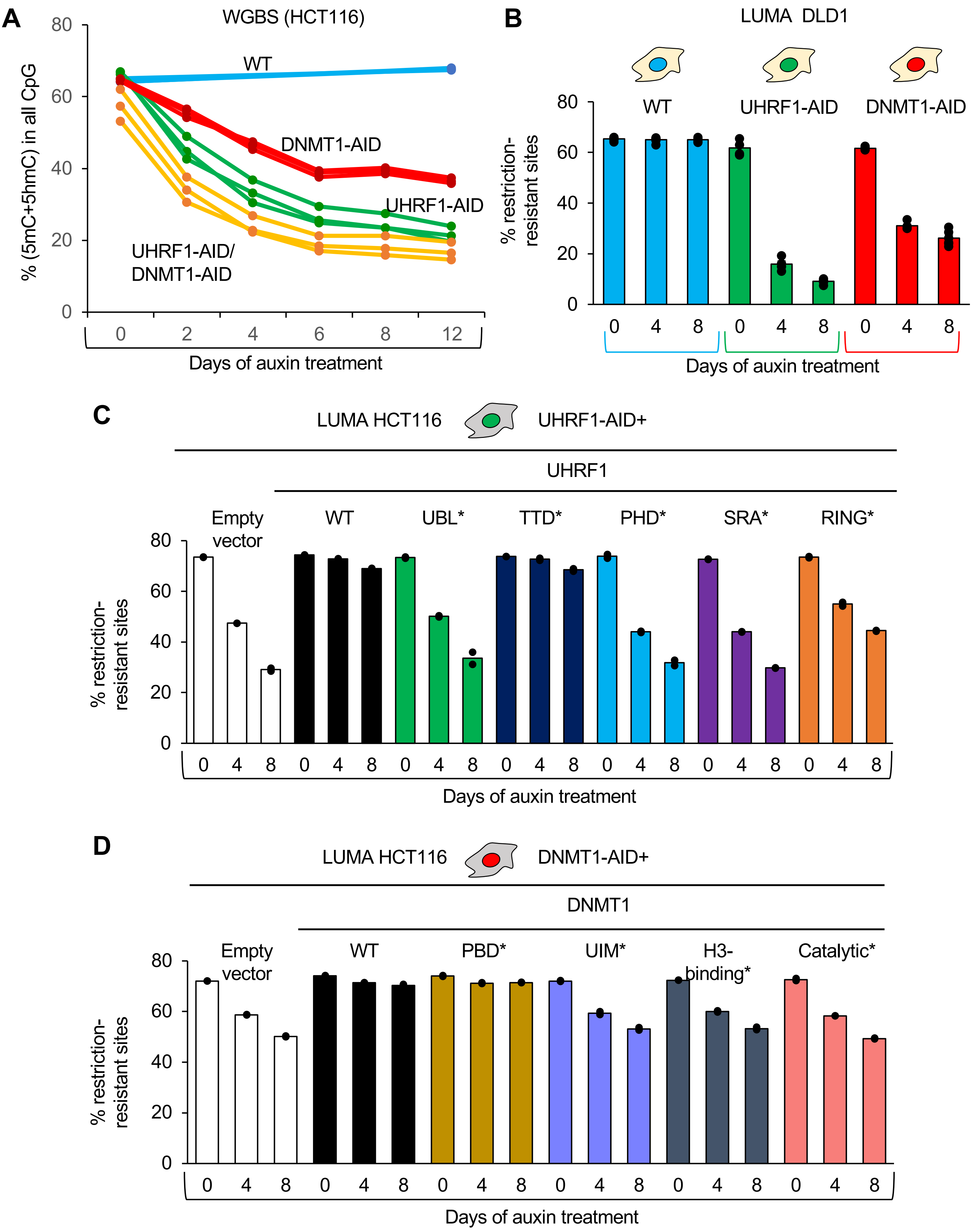

Supplementary Figure 3. Validation of the effects of UHRF1 and DNMT1 degradation on DNA methylation in DLD1 cells; identification of the domains essential for DNA methylation.
(A) WGBS on the indicated cell lines, with individual clones shown (n=3 each). (B) Global DNA methylation analysis in the indicated DLD1 derivatives after auxin treatment for the indicated duration (LUMA). Error bars represent the SEM of biological triplicates. (C) Global DNA methylation analysis in the indicated HCT116 UHRF1-AID rescue lines. (D) As in panel B, but for DNMT1-AID.

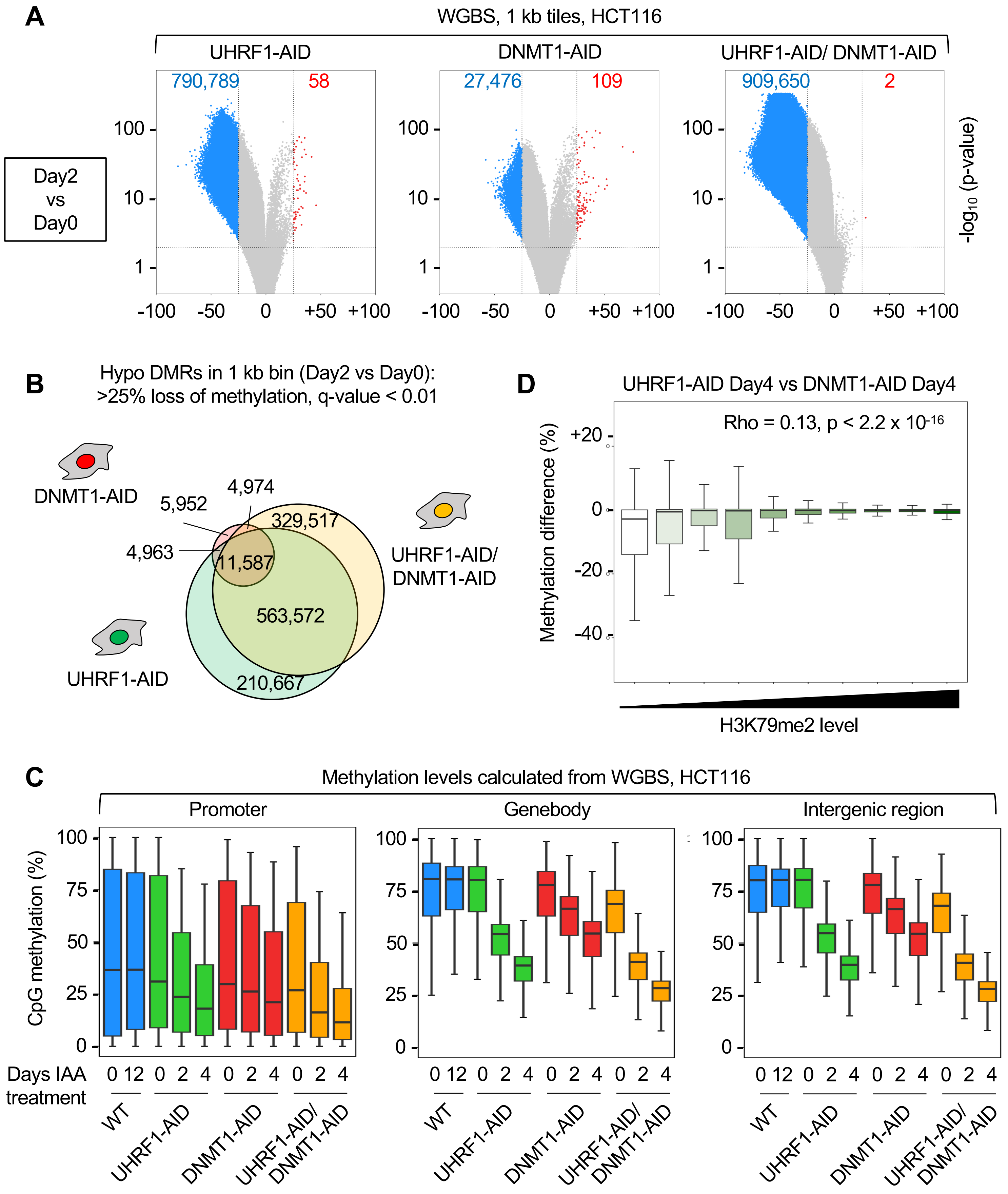

Supplementary Figure 4. Greater loss of DNA methylation upon UHRF1 depletion than upon DNMT1 depletion; additional data.

(A) Volcano plot of differentially methylated regions (DMRs, 1kb bins) after 2 days of depletion of UHRF1 and/or DNMT1. Blue dots: hypomethylated regions (>25% loss of methylation, q-value < 0.01), red dots: hypermethylated regions (>25% gain of methylation, q-value < 0.01), gray dots: no significant change. The p-value is corrected to q-value using sliding linear model (SLIM). (B) Venn diagram of the hypomethylated regions in the indicated cell lines, 2 days after depletion of the proteins. (C) Boxplots of CpG methylation (%) in the indicated regions and conditions. Promoters: from -1000 to + 200 bps from TSS; Gene bodies obtained from hg38 refFlat by removal of the promoter regions; Intergenic regions: the whole genome minus promoters and gene bodies. (D) Lack of correlation between H3K79me2 levels and DNA methylation loss at CpG islands; legend as in Panel 4D.

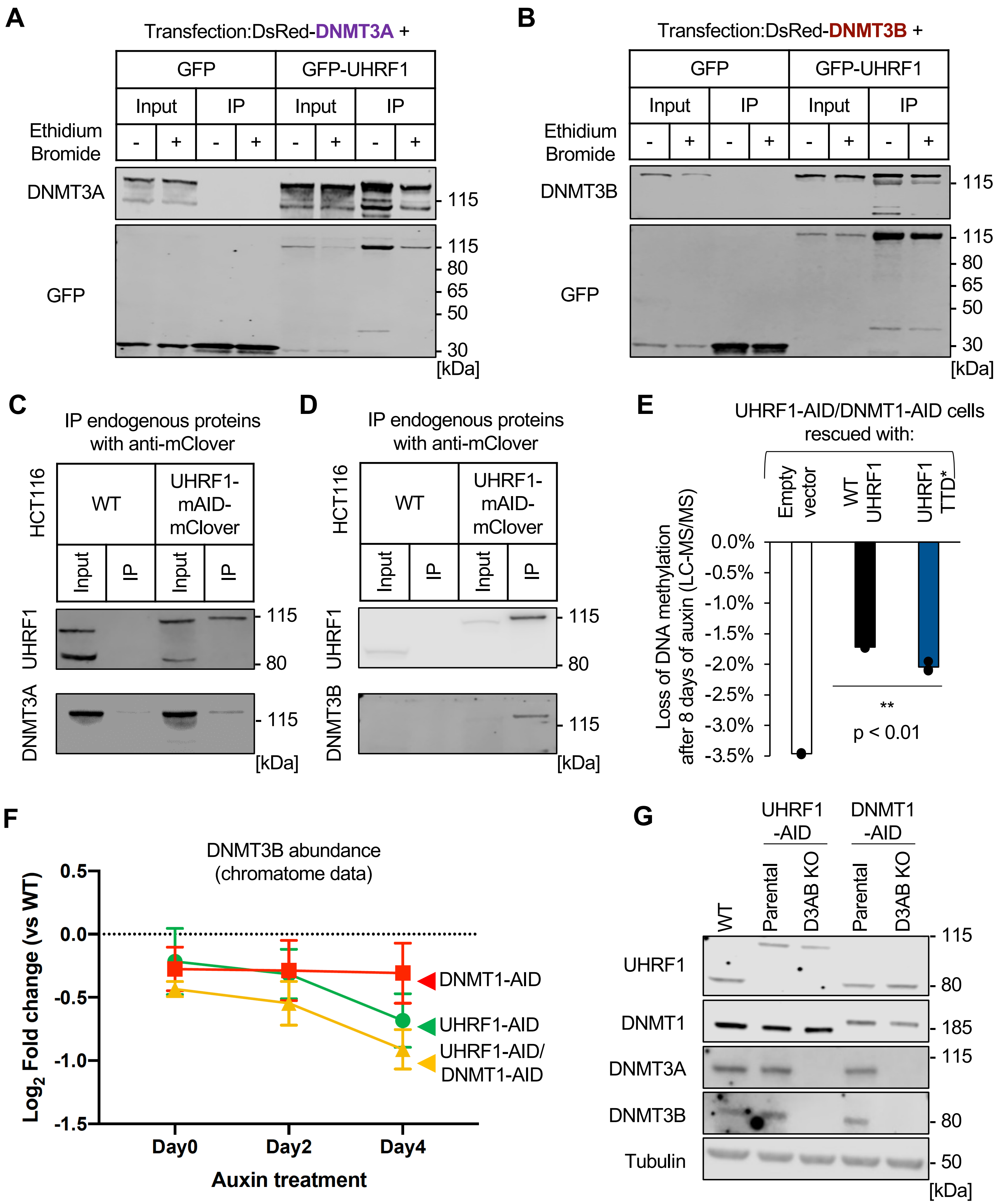

Supplementary Figure 5. Additional controls for the UHRF1/DNMT3A/DNMT3B interaction; chromatome experiments reveal the effect of UHRF1 depletion on DNMT3B; validation of the DNMT3A/DNMT3B KOs
(A-B) Western blotting after the indicated co-immunoprecipitation experiments, without or with Ethidium Bromide (20 μg/mL). (C) Co-immunoprecipitation of endogenous UHRF1 and DNMT3A. (D) Co-immunoprecipitation of endogenous UHRF1 and DNMT3B. (E) A TTD mutant form of UHRF1 only partially rescues UHRF1-AID/DNMT1-AID cells. Quantitation of the loss of DNA methylation in the indicated cell lines after 8 days of protein depletion, by LC-MS/MS. The p-value is calculated with one-way ANOVA and Tukey's HSD test (**$p < 0.01$). (F) Illustration of the chromatome results for DNMT3B, which is less abundant upon UHRF1 depletion. (G) Validation by western blotting of the DNMT3A and DNMT3B CRISPR KOs in UHRF1-AID or DNMT1-AID HCT116 cells. (A-D, G) Experiments in each panel were performed at least two times, and the representative results are shown.

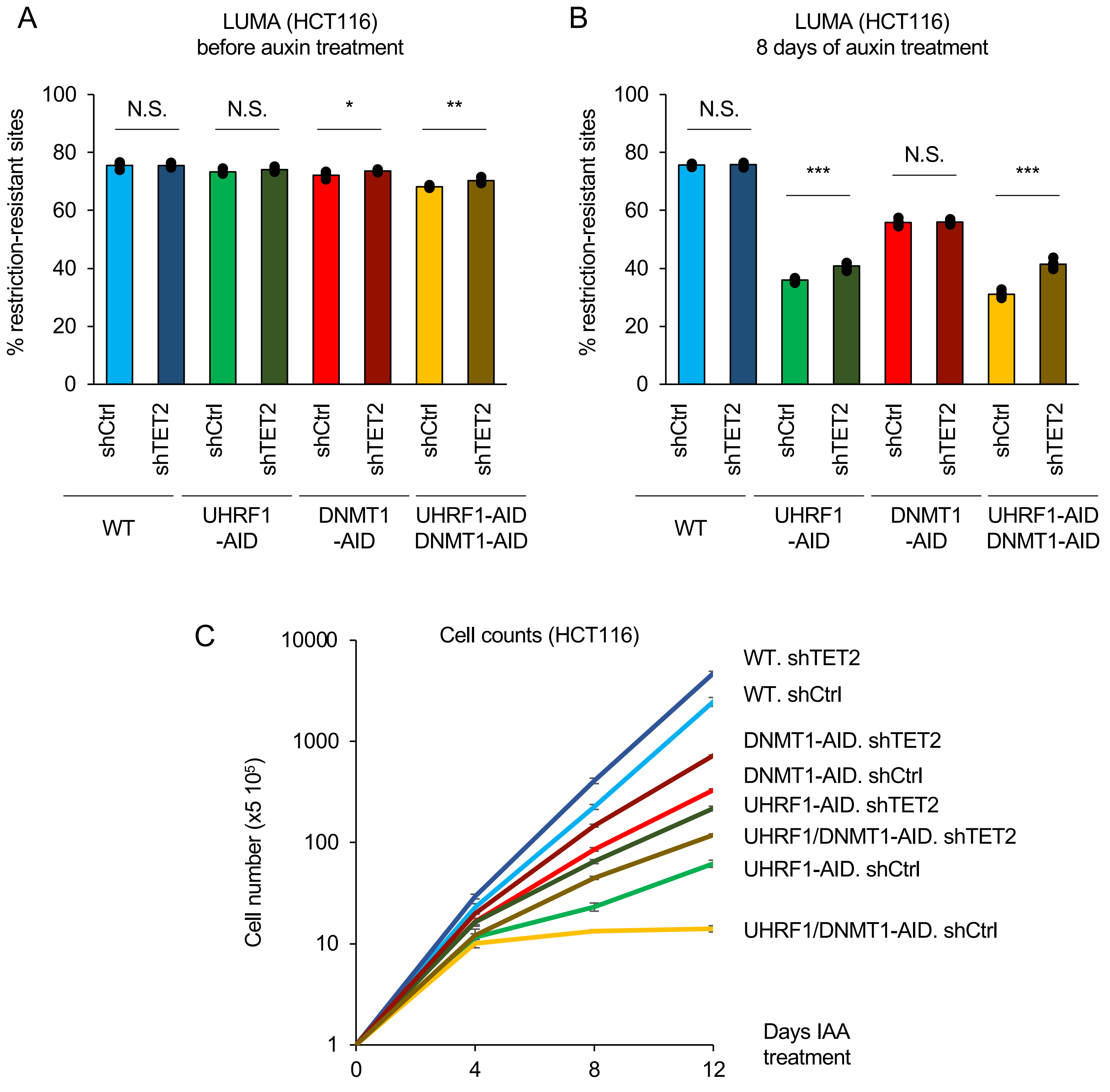

Supplementary Figure 6. UHRF1 protects against active demethylation by TET2: additional data and controls. (A) Global DNA methylation analysis (LUMA) for HCT116 UHRF1 and/or DNMT1-AID cell lines combined with TET2 knockdown, in the absence of auxin. Error bars represent the SEM of 3 independent experiments. The p-value is calculated using two-sided Student's t-test (N.S. $p>0.05$, *$p < 0.05$, **$p < 0.01$, ***$p < 0.001$). (B) As in Panel A, but following 8 days of auxin treatment. (C) Growth curves of the indicated cell lines, in the presence of auxin (Cell counts). Error bars represent the SEM of 3 independent experiments.

Supplementary Table 1. Summary of the primers and oligonucleotide sequences in this study.

Name	Strand	Sequence (5' - 3')	Purpose
DNMT3A-KO	F	caccgCGATGACGAGCCAGAGTACG	CRISPR-KO for DNMT3A
	R	aaacCGTACTCTGGCTCGTCATCGc	
DNMT3B-KO1	F	caccgATCCGCACCCCGGAGATCAG	CRISPR-KO for DNMT3B
	R	aaacCTGATCTCCGGGGTGCGGATc	
DNMT3B-KO2	F	caccgAGAGTCGCGAGCTTGATCTT	CRISPR-KO for DNMT3B
	R	aaacAAGATCAAGCTCGCGACTCTc	
shTET2	F	ccggTTTCACGCCAAGTCGTTATTTctcgagAAATAACGACTTGGCGTGAAAtttttg	TET2 knockdown
	R	aattcaaaaaTTTCACGCCAAGTCGTTATTTctcgagAAATAACGACTTGGCGTGAAA	
shCtrl	F	ccggCAACAAGATGAAGAGCACCAActcgagTTGGTGCTCTTCATCTTGTTGtttttg	Non-targeting shRNA
	R	aattcaaaaaCAACAAGATGAAGAGCACCAActcgagTTGGTGCTCTTCATCTTGTTG	
TET2	F	GCTTACCGAGACGCTGAGGAAA	TET2 RT-qPCR
	R	AGAGAAGGAGGCACCACAGGTT	
TBP1	F	TGGCCCATAGTGATCTTTGC	TBP1 RT-qPCR for internal control
	R	TCCTAGAGCATCTCCAGCACA	
PGK1	F	AGGATAAAGTCAGCCATGTGAG	PGK1 RT-qPCR for internal control
	R	CACAGGAACTAAAAGGCAGGA	

Supplementary Table 2. Summary of WGBS basic metrics.

**Summary of mapping**	Total PE1 reads	Uniquely mapped PE1	Unmapped PE1	Total PE2 reads	Uniquely mapped PE2	Unmapped PE2
WT.Day0	65,093,997	87.60%	7.70%	65,093,997	84.90%	10.60%
WT.Day12	104,566,542	85.80%	9.40%	104,566,542	80.90%	14.60%
Uaid.Day0	389,406,712	87.40%	7.90%	389,406,712	84.60%	10.80%
Uaid.Day2	399,761,323	86.40%	8.70%	399,761,321	82.70%	12.70%
Uaid.Day4	451,085,724	85.70%	9.30%	451,085,724	82.00%	13.20%
Uaid.Day6	77,194,669	85.90%	9.20%	77,194,667	81.00%	14.40%
Uaid.Day8	94,550,101	85.70%	9.30%	94,550,096	80.80%	14.60%
Uaid.Day12	130,497,852	85.80%	9.30%	130,497,849	80.60%	14.80%
Daid.Day0	389,637,848	87.20%	8.00%	389,637,843	84.30%	11.20%
Daid.Day2	401,146,513	86.10%	9.10%	401,146,508	81.90%	13.60%
Daid.Day4	406,630,131	85.80%	9.20%	406,630,129	82.00%	13.30%
Daid.Day6	75,840,116	85.80%	9.30%	75,840,114	80.90%	14.50%
Daid.Day8	95,647,106	85.70%	9.40%	95,647,107	80.90%	14.40%
Daid.Day12	116,240,399	85.70%	9.40%	116,240,399	80.30%	15.10%
UDaid.Day0	391,251,162	85.90%	9.20%	391,251,162	82.20%	13.20%
UDaid.Day2	406,371,889	85.90%	9.10%	406,371,886	81.70%	13.60%
UDaid.Day4	411,497,719	85.60%	9.30%	411,497,718	81.50%	13.80%
UDaid.Day6	84,256,670	85.80%	9.20%	84,256,670	81.00%	14.30%
UDaid.Day8	102,849,469	85.60%	9.40%	102,849,470	81.00%	14.30%
UDaid.Day12	116,889,481	85.70%	9.30%	116,889,480	80.60%	14.70%
HCT116.WT	414,566,069	86.20%	9.00%	414,566,067	83.70%	11.80%
HCT116.dKO	31,994,368	87.10%	8.00%	31,994,371	84.20%	11.20%

**Summary of mapping**	Mean depth				
	All N	Aall C	All CpG	All CHG	All CHH
WT.Day0	2.1	2.2	2	2.3	2.2
WT.Day12	3.4	3.7	3.7	3.7	3.6
Uaid.Day0	12.5	13.1	12.1	13.4	13.3
Uaid.Day2	12.8	13.5	13.5	13.8	13.6
Uaid.Day4	14.3	15.2	15.6	15.7	15.3
Uaid.Day6	2.5	2.7	2.8	2.7	2.7
Uaid.Day8	3.1	3.3	3.3	3.3	3.3
Uaid.Day12	4.3	4.6	4.7	4.7	4.5
Daid.Day0	12.5	13	12.1	13.3	13.2
Daid.Day2	12.7	13.5	13.5	13.8	13.5
Daid.Day4	13	13.9	14.3	14.2	13.9
Daid.Day6	2.5	2.6	2.7	2.7	2.6
Daid.Day8	3.1	3.3	3.3	3.4	3.3

Daid.Day12	3.8	4	4.2	4.1	4
UDaid.Day0	12.6	13.3	13.5	13.7	13.4
UDaid.Day2	12.9	13.7	14.1	14.1	13.8
UDaid.Day4	13.2	14.1	14.5	14.4	14.1
UDaid.Day6	2.7	2.9	3	3	2.9
UDaid.Day8	3.3	3.6	3.6	3.6	3.6
UDaid.Day12	3.8	4.1	4.2	4.2	4.1
HCT116.WT	13.4	14.3	14.6	14.8	14.3
HCT116.dKO	1	1.1	1.1	1.1	1.1

**Summary of mapping**	Methylation level			
	All C	All CpG	All CHG	All CHH
WT.Day0	3.5	66.5	1	1
WT.Day12	4.1	68.9	1.1	1
Uaid.Day0	4	68.5	1.1	1
Uaid.Day2	3.2	46.9	1.2	1.1
Uaid.Day4	2.9	34.8	1.4	1.4
Uaid.Day6	2.4	27.3	1.3	1.2
Uaid.Day8	2.1	25.6	1	1
Uaid.Day12	2.1	22	1.2	1.2
Daid.Day0	4	66.6	1.2	1.1
Daid.Day2	3.7	57	1.2	1.1
Daid.Day4	3.4	47.3	1.4	1.3
Daid.Day6	2.8	39.7	1.1	1.1
Daid.Day8	2.7	40.4	1	1
Daid.Day12	2.8	37.3	1.3	1.2
UDaid.Day0	3.8	58.6	1.3	1.2
UDaid.Day2	2.7	35	1.2	1.2
UDaid.Day4	2.2	24.7	1.2	1.1
UDaid.Day6	1.9	19.4	1.1	1
UDaid.Day8	1.9	18.9	1.1	1
UDaid.Day12	1.9	17.2	1.2	1.1
HCT116.WT	4.3	71.6	1.1	1
HCT116.dKO	2.5	33	1.1	1

**Summary of mapping**	Coverage vs read depth			
	1 (all N)	3 (all N)	5 (all N)	10 (all N)
WT.Day0	67.10%	34.30%	13.60%	0.80%
WT.Day12	76.00%	53.00%	30.30%	4.20%
Uaid.Day0	83.00%	80.10%	76.30%	60.20%
Uaid.Day2	83.20%	80.60%	77.10%	61.40%

Uaid.Day4	83.50%	81.30%	78.50%	65.80%
Uaid.Day6	71.10%	40.60%	18.10%	1.30%
Uaid.Day8	74.60%	49.00%	25.80%	2.80%
Uaid.Day12	78.50%	61.00%	40.30%	8.30%
Daid.Day0	83.10%	80.30%	76.50%	60.20%
Daid.Day2	83.30%	80.70%	77.20%	61.40%
Daid.Day4	83.30%	80.80%	77.40%	61.90%
Daid.Day6	70.70%	39.90%	17.60%	1.30%
Daid.Day8	74.90%	49.60%	26.40%	2.90%
Daid.Day12	77.30%	56.90%	34.80%	5.70%
UDaid.Day0	83.20%	80.60%	77.00%	60.60%
UDaid.Day2	83.30%	80.80%	77.50%	62.10%
UDaid.Day4	83.30%	80.90%	77.60%	62.60%
UDaid.Day6	72.50%	44.20%	21.50%	2.00%
UDaid.Day8	75.70%	52.20%	29.20%	3.80%
UDaid.Day12	77.30%	57.00%	35.10%	5.90%
HCT116.WT	83.20%	80.60%	77.10%	62.10%
HCT116.dKO	48.50%	13.40%	2.90%	-
