# Supplementary material for: Non-canonical functions of UHRF1 maintain DNA methylation homeostasis in cancer cells
Source: Nat Commun. 2024 Apr 5;15:2960. doi: 10.1038/s41467-024-47314-4 (PMC10997609; doi:10.1038/s41467-024-47314-4)
Supplement: Supplementary file 3 — Description of Additional Supplementary Files [file 41467_2024_47314_MOESM3_ESM.pdf]

Description of Additional Supplementary Files

File Name: Supplementary Movie 1
Description: Live cell imaging for UHRF1 and DNMT1 with HCT116 UHRF1-AID/ DNMT1-AID cell line. Only mClover (UHRF1) signal is displayed in this movie. Timelapse images were taken every 10 minutes for 20 hours.

File Name: Supplementary Movie 2
Description: Live cell imaging for UHRF1 and DNMT1 with HCT116 UHRF1-AID/ DNMT1-AID cell line. Only mRuby (DNMT1) signal is displayed in this movie. Timelapse images were taken every 10 minutes for 20 hours.

File Name: Supplementary Movie 3
Description: Live cell imaging for UHRF1 and DNMT1 with HCT116 UHRF1-AID/ DNMT1-AID cell line. In this movie, mClover (UHRF1) signal is displayed as green and mRuby (DNMT1) signal is displayed as red. Colocalizing foci are formed during S phase. Timelapse images were taken every 10 minutes for 20 hours.
