# Supplementary material for: Non-canonical functions of UHRF1 maintain DNA methylation homeostasis in cancer cells
Source: Nat Commun. 2024 Apr 5;15:2960. doi: 10.1038/s41467-024-47314-4 (PMC10997609; doi:10.1038/s41467-024-47314-4)
Supplement: Supplementary file 7 — Reporting Summary [file 41467_2024_47314_MOESM7_ESM.pdf]

nature portfolio

Corresponding author(s): Kosuke Yamaguchi
Pierre-Antoine Defossez

Last updated by author(s): Feb 22, 2024

Reporting Summary

Nature Portfolio wishes to improve the reproducibility of the work that we publish. This form provides structure for consistency and transparency in reporting. For further information on Nature Portfolio policies, see our Editorial Policies and the Editorial Policy Checklist.

Statistics

For all statistical analyses, confirm that the following items are present in the figure legend, table legend, main text, or Methods section.

n/a	Confirmed	
☐	☒	The exact sample size (*n*) for each experimental group/condition, given as a discrete number and unit of measurement
☐	☒	A statement on whether measurements were taken from distinct samples or whether the same sample was measured repeatedly
☐	☒	The statistical test(s) used AND whether they are one- or two-sided<br>*Only common tests should be described solely by name; describe more complex techniques in the Methods section.*
☒	☐	A description of all covariates tested
☐	☒	A description of any assumptions or corrections, such as tests of normality and adjustment for multiple comparisons
☐	☒	A full description of the statistical parameters including central tendency (e.g. means) or other basic estimates (e.g. regression coefficient) AND variation (e.g. standard deviation) or associated estimates of uncertainty (e.g. confidence intervals)
☐	☒	For null hypothesis testing, the test statistic (e.g. *F*, *t*, *r*) with confidence intervals, effect sizes, degrees of freedom and *P* value noted<br>*Give P values as exact values whenever suitable.*
☒	☐	For Bayesian analysis, information on the choice of priors and Markov chain Monte Carlo settings
☒	☐	For hierarchical and complex designs, identification of the appropriate level for tests and full reporting of outcomes
☐	☒	Estimates of effect sizes (e.g. Cohen's *d*, Pearson's *r*), indicating how they were calculated

Our web collection on statistics for biologists contains articles on many of the points above.

Software and code

Policy information about availability of computer code

Data collection

No software was used for collection of data

Data analysis

Statistics analysis:
R v4.1.0

Flow cytometry analysis:
FlowJo v10.6.2

Microscopy/ Videomicroscopy analysis:
ImageJ v2.1.0/1.53c

Sequencing data analysis:
FASTQC v0.11.9
Trimmomatic v0.38
STAR v2.6.1d
FeatureCounts v1.5.0-p3
DESeq2 v1.24.0
BMap v1.0
methylKit v1.20.0
bowtie2 v2.4.5
samtools v1.9

bedtools v2.29.2
R v4.1.0

For manuscripts utilizing custom algorithms or software that are central to the research but not yet described in published literature, software must be made available to editors and reviewers. We strongly encourage code deposition in a community repository (e.g. GitHub). See the Nature Portfolio guidelines for submitting code & software for further information.

Data

Policy information about availability of data

All manuscripts must include a data availability statement. This statement should provide the following information, where applicable:

- Accession codes, unique identifiers, or web links for publicly available datasets
- A description of any restrictions on data availability
- For clinical datasets or third party data, please ensure that the statement adheres to our policy

The WGBS and RNA-seq data has been submitted to GEO under references GSE236026 (WGBS, https://www.ncbi.nlm.nih.gov/geo/query/acc.cgi?acc=GSE236026) and GSE249536 (RNA-seq, https://www.ncbi.nlm.nih.gov/geo/query/acc.cgi?acc=GSE249536).

The mass spectrometry proteomics data have been deposited to the ProteomeXchange Consortium via the PRIDE70 partner repository with the dataset identifier PXD043254 (https://proteomecentral.proteomexchange.org/cgi/GetDataset?ID=PXD043254).

H3K36me3 and H3K79me2 ChIP-seq data used in this study were obtained from ENCODE project (https://www.encodeproject.org/). The accession numbers are ENCSR091QXP (H3K36me3) and ENCSR494CCN (H3K79me2).

Source data are provided with this paper.

Research involving human participants, their data, or biological material

Policy information about studies with human participants or human data. See also policy information about sex, gender (identity/presentation), and sexual orientation and race, ethnicity and racism.

Reporting on sex and gender	Sex and gender information have not been collected in this paper. Sex was not considered in the study design
Reporting on race, ethnicity, or other socially relevant groupings	The reporting on race, ethnicity, or other socially relevant groupings data is not used in this paper.
Population characteristics	The population characteristics are not used in this paper.
Recruitment	This paper does not include the data from recruitment of patient or volunteer.
Ethics oversight	This paper does not include human participants data to require ethics oversight.

Note that full information on the approval of the study protocol must also be provided in the manuscript.

Field-specific reporting

Please select the one below that is the best fit for your research. If you are not sure, read the appropriate sections before making your selection.

☒ Life sciences ☐ Behavioural & social sciences ☐ Ecological, evolutionary & environmental sciences

For a reference copy of the document with all sections, see nature.com/documents/nr-reporting-summary-flat.pdf

Life sciences study design

All studies must disclose on these points even when the disclosure is negative.

Sample size

For statistical analysis in this study, we used n=3 with biological replicates or independent experiments.
For biological replicates, below cell lines are prepared from 3 different clones to avoid cloning biases.
- UHRF1-AID (HCT116/ DLD1)
- DNMT1-AID (HCT116/ DLD1)
- UHRF1-AID/DNMT1-AID (HCT116)
- DNMT3A/3B DKO in UHRF1-AID (HCT116)
- DNMT3A/3B DKO in DNMT1-AID (HCT116)

Related to rescue (Figure 2E and F) and knockdown (Figure 6) experiments, we selected infected cells with antibiotic marker from bulk population to avoid cloning biases, and confirmed results with three independent experiments.

For non-statistical analysis in this study, we confirmed similar results with at least two independent experiments.

Data exclusions	No data was excluded.
Replication	We confirmed that all attempts to replicate experiments were successful. The replicated experiments and analyses are:<br>- cell lines validation for AID tagging and protein degradation, 3x biological replicates<br>- live cell imaging analysis, 3x biological replicates<br>- microscopy analysis for UHRF1 and DNMT1 co-localization, 3x independent experiments<br>- measurement of DNA methylation by LUMA and mass spectrometry in each AID cell lines, 3x biological replicates<br>- WGBS analysis for each cell lines and time points, 3x biological replicates<br>- RNA-seq analysis for each cell lines and time points, 3x biological replicates<br>- cell proliferation and viability analysis in each AID cell lines, 3x biological replicates<br>- flow cytometry analysis in each AID cell lines, 3x biological replicates<br>- rescue experiments with exogenous UHRF1 or DNMT1 for cell proliferation assay, 3x independent experiments<br>- measurement of DNA methylation by LUMA and protein degradation after 5-AZA treatment, 2x independent experiments<br>- co-immunoprecipitation assay, 2x independent experiments<br>- measurement of DNA methylation by mass spectrometry in DNMT3s KO cell lines, 3x biological replicates<br>- TET2 knockdown validation with RT-qPCR, 3x independent experiments<br>- measurement of DNA methylation by LUMA and mass spectrometry in TET2 knockdown samples, 3x independent experiments
Randomization	HCT116 and DLD1 AID cell lines were selected randomly to make single clone cell lines. Related to rescue (Figure 2E and F) and knockdown (Figure 6) experiments, infected cells were selected randomly with antibiotic marker from bulk population. Randomization of other experiments was not relevant to this study.
Blinding	Blinding was not relevant to data analysis. The all signal and images related to this study are measured by automated method, and the authors did not do any manual measurement.

Reporting for specific materials, systems and methods

We require information from authors about some types of materials, experimental systems and methods used in many studies. Here, indicate whether each material, system or method listed is relevant to your study. If you are not sure if a list item applies to your research, read the appropriate section before selecting a response.

Materials & experimental systems

n/a	Involved in the study	
☐	☒	Antibodies
☐	☒	Eukaryotic cell lines
☒	☐	Palaeontology and archaeology
☒	☐	Animals and other organisms
☒	☐	Clinical data
☒	☐	Dual use research of concern
☒	☐	Plants

Methods

n/a	Involved in the study	
☒	☐	ChIP-seq
☐	☒	Flow cytometry
☒	☐	MRI-based neuroimaging

Antibodies

Antibodies used	Santa Cruz Biotechnology: sc-98817 for UHRF1 (1:1,000 for WB)<br>Cell Signaling Technology; #5032 for DNMT1 (1:1,000 dilution for WB)<br>Abcam: ab7291 for Tubulin (1:1,000 dilution for WB)<br>Abcam: ab188470 for DNMT3A (1:1,000 dilution for WB)<br>Cell Signaling Technology: 67259T for DNMT3B (1:1,000 dilution for WB)<br>Roche: 11 814 460 001 for GFP (1:1,000 dilution for WB)
Validation	All antibodies are purchased and validated in each company.<br>Rabbit Anti-UHRF1 for WB (https://datasheets.scbt.com/sc-98817.pdf)<br>Rabbit Anti-DNMT1 for WB (https://www.cellsignal.com/products/primary-antibodies/dnmt1-d63a6-xp-rabbit-mab/5032)<br>Mouse Anti-Tubulin for WB (https://www.abcam.co.jp/products/primary-antibodies/alpha-tubulin-antibody-dm1a-loading-control-ab7291.html)<br>Rabbit Anti-DNMT3A for WB (https://www.abcam.co.jp/products/primary-antibodies/dnmt3a-antibody-epr18455-ab188470.html)<br>Rabbit Anti-DNMT3B for WB (https://www.cellsignal.com/products/primary-antibodies/dnmt3b-d7o7o-rabbit-mab/67259)<br>Mouse Anti-GFP for WB (https://www.sigmaaldrich.com/FR/fr/product/roche/11814460001)

Eukaryotic cell lines

Policy information about cell lines and Sex and Gender in Research

Cell line source(s)	HCT116 cell line (Tet-inducible OsTIR1) was obtained form RIKEN BRC Cell Bank. DLD1 cell line (constitutiely expressing OsTIR1- F74G) was generated from MTK lab. AID tagged cell lines, exsogenous UHRF1 expressing cell lines, exogenous DNMT1 expressing cell lines, DNMT3A and DNMT3B knockout cell lines, and TET2 knockdown cell lines were generated in PAD lab during the course of this study.

Authentication	AID tagging UHRF1 and DNMT1 was confirmed by PCR, western blot, and sanger sequence. Knockout for DNMT3A and DNMT3B was validated by western blot. Knockdown for TET2 was validated by RT-qPCR. Parent cell lines (HCT116 and DLD1) were authenticated by STR profiling.
Mycoplasma contamination	All cell lines tested negative for mycoplasma.
Commonly misidentified lines (See ICLAC register)	No commonly misidentified cell lines were used in this study.

Flow Cytometry

Plots

Confirm that:

☐ The axis labels state the marker and fluorochrome used (e.g. CD4-FITC).

☐ The axis scales are clearly visible. Include numbers along axes only for bottom left plot of group (a 'group' is an analysis of identical markers).

☐ All plots are contour plots with outliers or pseudocolor plots.

☒ A numerical value for number of cells or percentage (with statistics) is provided.

Methodology

Sample preparation	WT, UHRF1-AID, DNMT1-AID, and UHRF1/DNMT1-AID HCT116 cells were incubated with 10 µM 5-bromo-2'-deoxyuridine (BrdU) for 45 min at 37°C in a CO_2 incubator. The cells were trypsinized and collected in a 15 mL tube. After washing with PBS, the cell pellet was resuspended in 750 µL PBS, then 2250 µL ice-cold ethanol added to fix the cells (3 mL final volume of 75% ethanol). Fixed cells were incubated for at least 30 min at -20 °C, and stored before performing flow cytometry analysis. Cellular DNA was denaturated in 2N HCl for 15 min, followed by pelleting the fixed cells and washing with PBS + 1% BSA. BrdU was then detected with the mouse anti-BrdU-FITC antibody (BD Biosciences) in PBS + 1% BSA. For cell cycle analysis, cells were rinsed with PBS + 1% BSA, then resuspended in PBS containing propidium iodide (1:500, Invitrogen) and 150 µg/mL RNaseA. Cells were incubated overnight at 4°C in the dark.
Instrument	The percentages of cells in subG1, G1, S and G2/M phases were measured with FACSCalibur (BD Biosciences).
Software	Data were analyzed with the FlowJo software (v10.6.2).
Cell population abundance	We described S phase cells as BrdU signal positive cells. The subG1, G1, and G2/M phases cells are described as BrdU signal negative cells. We separated G1 and G2/M phases cells with DNA contents (G1=2N, G2/M=4N). Sub G1 phase cells was described as lower DNA contents than G1 phase cells.
Gating strategy	The population was gated on size using SSC-A/FSC-A. To obtain single cell information, we used FSC-H/ FSC-A gating. All gated information was supplied for cell population analysis.

☒ Tick this box to confirm that a figure exemplifying the gating strategy is provided in the Supplementary Information.
